# Loss of the EPH receptor B6 contributes to colorectal cancer metastasis

**DOI:** 10.1038/srep43702

**Published:** 2017-03-06

**Authors:** Silvia Mateo-Lozano, Sarah Bazzocco, Paulo Rodrigues, Rocco Mazzolini, Elena Andretta, Higinio Dopeso, Yolanda Fernández, Edgar del Llano, Josipa Bilic, Lucía Suárez-López, Irati Macaya, Fernando Cartón-García, Rocio Nieto, Lizbeth M. Jimenez-Flores, Priscila Guimarães de Marcondes, Yaiza Nuñez, Elsa Afonso, Karina Cacci, Javier Hernández-Losa, Stefania Landolfi, Ibane Abasolo, Santiago Ramón y Cajal, John M. Mariadason, Simo Schwartz, Toshimitsu Matsui, Diego Arango

**Affiliations:** 1 Group of Biomedical Research in Digestive Tract Tumors, CIBBIM-Nanomedicine, Vall d’Hebron University Hospital, Research Institute (VHIR), Universitat Autònoma de Barcelona, Barcelona, Spain; 2 CIBER de Bioingeniería, Biomateriales y Nanomedicina (CIBER-BBN), Spain; 3 Group of Drug Delivery and Targeting, CIBBIM-Nanomedicine, Vall d’Hebron University Hospital, Research Institute (VHIR), Universitat Autònoma de Barcelona, Barcelona, Spain; 4 Functional Validation & Preclinical Research (FVPR), Vall d’Hebron University Hospital, Research Institute (VHIR), Universitat Autònoma de Barcelona, Barcelona, Spain; 5 Department of Pathology, Vall d’Hebron Hospital, Barcelona, Spain (CIBERONC); 6 Olivia Newton-John Cancer Research Institute and School of Cancer Medicine, La Trobe University, Melbourne, Australia; 7 Hematology, Department of Medicine, Kobe University Graduate School of Medicine, Chuo-ku, Kobe, Japan

## Abstract

Although deregulation of EPHB signaling has been shown to be an important step in colorectal tumorigenesis, the role of EPHB6 in this process has not been investigated. We found here that manipulation of EPHB6 levels in colon cancer cell lines has no effect on their motility and growth on a solid substrate, soft agar or in a xenograft mouse model. We then used an *EphB6* knockout mouse model to show that *EphB6* inactivation does not efficiently initiate tumorigenesis in the intestinal tract. In addition, when intestinal tumors are initiated genetically or pharmacologically in *EphB6*^+/+^ and *EphB6*^−/−^ mice, no differences were observed in animal survival, tumor multiplicity, size or histology, and proliferation of intestinal epithelial cells or tumor cells. However, reintroduction of EPHB6 into colon cancer cells significantly reduced the number of lung metastasis after tail-vein injection in immunodeficient mice, while EPHB6 knockdown in EPHB6-expressing cells increased their metastatic spread. Consistently, although EPHB6 protein expression in a series of 130 primary colorectal tumors was not associated with patient survival, EPHB6 expression was significantly lower in lymph node metastases compared to primary tumors. Our results indicate that the loss of EPHB6 contributes to the metastatic process of colorectal cancer.

Erythropoietin-Producing Hepatoma (EPH) receptors constitute the largest known family of receptor tyrosine kinases characterized in humans. These receptors and their Ephrin ligands are important for recognizing signals from the extracellular environment and are involved in cell-cell interaction, participating in cell adhesion, migration and proliferation. Consistent with these roles, several members of the EPH family have been shown to be involved in tumorigenesis in different organs^1^,^2^,^3^,^4^,^5^,^6^. Some EPH receptors, namely EPHA10 and EPHB6, lack kinase activity due to the presence of key amino acid changes in the kinase domain^7^,^8^. EPHB6 is widely expressed in multiple adult tissues^7^ and forms heterodimers with other EPH receptors to participate in signal transduction despite the lack of intrinsic kinase activity^9^,^10^. In line with the biological functions of other EPH receptors, EPHB6 has been shown to regulate cell adhesion and migration^11^,^12^,^13^.

The importance of B-type EPH receptors in controlling proliferation and the architecture of the normal intestinal epithelium has been extensively documented. EPHB2 and EPHB3 are important for maintaining the separation between proliferative and differentiating compartments in the normal intestinal epithelium^14^. Moreover, inactivation of EPHB2 and EPHB3 signaling reduces the number of proliferating cells in intestinal crypts by >50%^15^. Importantly, the loss of EPHB signaling has been reported to accelerate the tumorigenic process in the intestine. The loss of EphB2 and EphB3 is associated with the adenoma-to-carcinoma transition and significantly contributes to intestinal tumor progression in animal models^6^. Over 40% of the gastrointestinal tumors showing a microsatellite instable (MSI) phenotype have inactivating mutations of *EPHB2*^2^,^4^ and low levels of EPHB2 in primary colorectal tumors are associated with poor patient prognosis^16^. In addition, EPHB4 expression has also been shown to be frequently lost in colorectal tumors^3^,^6^ and low EPHB4 levels are associated with shorter patient survival^3^. Moreover, inactivation of a single allele of *EphB4* in mice is sufficient to significantly accelerate intestinal tumorigenesis^5^. However, the role of EPHB6 in colorectal cancer has not been investigated.

EPHB6 has been reported to be frequently mutated in melanomas (7/79, 8.9%)^17^, with particularly high incidence in desmoplastic melanomas (17/62, 27.4%)^18^, although the biological significance of these changes has not been investigated. However, the loss of EPHB6 has been shown to significantly contribute to the metastatic spread of several tumor types, including breast and lung tumors^19^,^20^. Here, we have investigated the importance of the loss of EPHB6 during intestinal tumorigenesis using isogenic *in vitro* systems, different mouse models and annotated collections of primary colorectal tumors, and found that the loss of EPHB6 contributes to the metastatic spread of colorectal tumors.

## Results

### EPHB6 does not regulate the motility, anchorage dependence or growth of colon cancer cells

**Figure 1:**
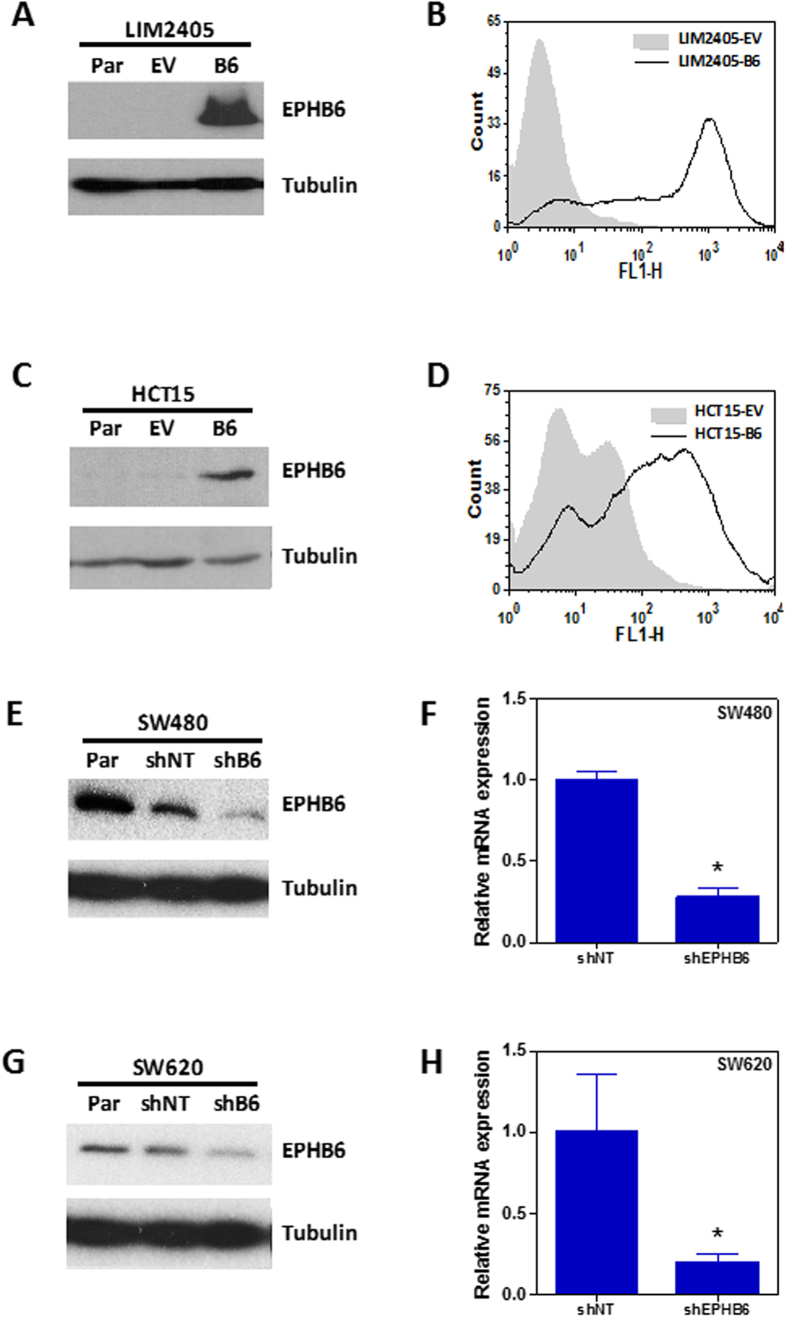
Engineered isogenic cell systems. EPHB6 was stably overexpressed into two colon cancer cell lines with low endogenous EPHB6 levels. Expression levels were confirmed by Western blotting (**A** and **C**) and flow cytometry (**B** and **D**) in LIM2405 (**A** and **B**) and HCT15 (**C** and **D**) cells. Par: parental; EV: empty vector; B6: EPHB6-HA. SW620 and SW480 colon cancer cells were stably transduced with an EPHB6 specific shRNA or a non-target (NT) shRNA. Reduced expression was confirmed at the protein level by Western blotting (**E** and **G**) and at the mRNA level by qPCR (**F** and **H**). The mean ± SEM is shown in panels **F** and **G**. Asterisks indicate p < 0.0001 (Student’s t-test).

Because EPHB signaling through other family members has been previously shown to be important in colorectal tumorigenesis^5^,^6^ we decided to investigate the role of EPHB6 in colorectal carcinogenesis using isogenic *in vitro* systems. First, we assessed the levels of EPHB6 mRNA and protein expression in a panel of 25 colorectal cancer cell lines. The majority of these cell lines showed low or undetectable expression of EPHB6 (22/25; 88.0% at the protein level; Supplementary Figure 1). We selected two cell lines with undetectable EPHB6 protein expression (LIM2405 and HCT15; Supplementary Figure 1) and stably overexpressed EPHB6. The overexpression of EPHB6 was confirmed by Western blotting and the membrane localization of the ectopically expressed EPHB6 was confirmed by flow cytometry (Fig. 1A–D). In addition, we selected two cell lines with high EPHB6 protein expression (SW480 and SW620; Supplementary Figure 1) and stably knocked down the expression of this EPH receptor. Reduced EPHB6 expression was confirmed both at the mRNA and protein level (Fig. 1E–H).

**Figure 2:**
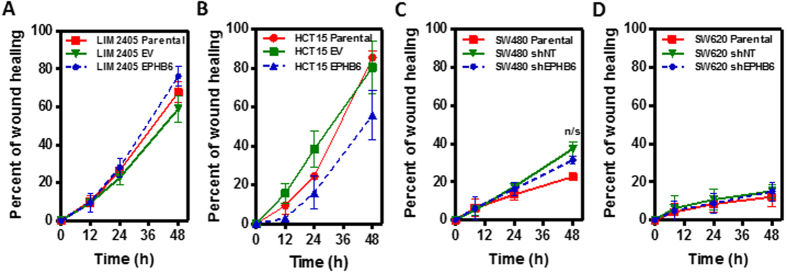
EPHB6 does not regulate cell motility/migration of colon cancer cells. A wound healing assay was used to assess the effects of EPHB6 overexpression on cell migration after EPHB6 overexpression in LIM2405 (**A**) and HCT115 (**B**) cells or EPHB6 knockdown in SW480 (**C**) and SW620 (**D**). The quantification of three independent experiments carried out in triplicate is shown (mean ± SEM). EV: empty vector; B6: EPHB6-HA; shNT: non-target shRNA; shEPHB6: EPHB6 targeting shRNA; n/s: p > 0.05 (Student’s t-test).

**Figure 3:**
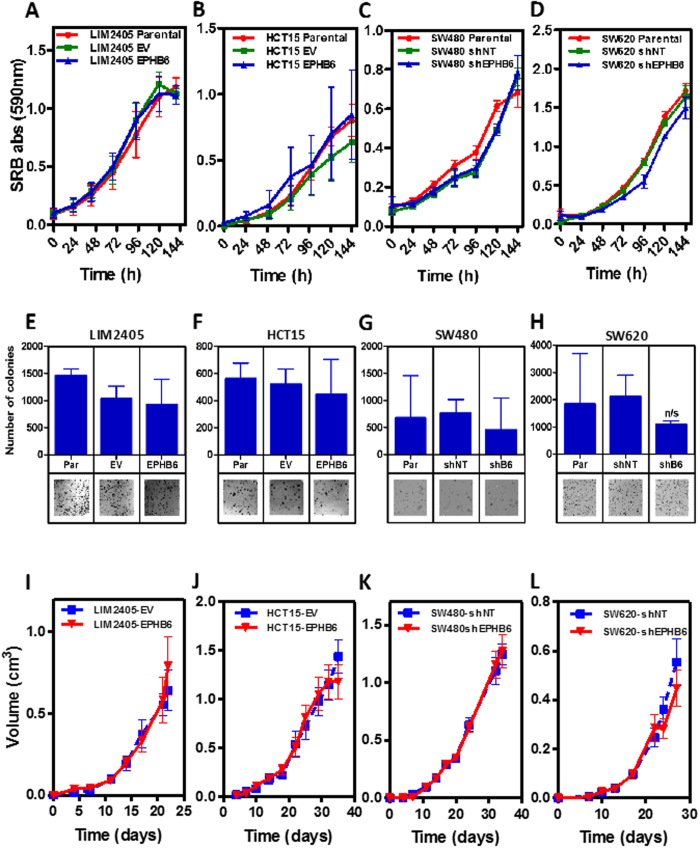
EPHB6 does not regulate the growth of colon cancer cells *in vitro*. Sulforhodamine B (SRB) staining was used to calculate the doubling time of colon cancer cell lines after manipulation of EPHB6 levels. Overexpression of EPHB6 in LIM2405 (**A**) and HCT15 (**B**) or EPHB6 knockdown in SW480 (**C**) and SW620 (**D**) had no effects on the growth of these cells *in vitro*. The mean of three different experiments (±SEM) is shown. A soft-agar colony formation assay was used to assess differences in anchorage-independent growth as a function of EPHB6 levels. Overexpression of EPHB6 in LIM2405 (**E**) and HCT15 (**F**) or EPHB6 knockdown in SW480 (**G**) and SW620 (**H**) did not affect their anchorage-independent growth. Histograms show the quantification of three independent experiments carried out in triplicate (mean ± SEM; n/s: p > 0.05, Student’s t-test). The pictures under the histograms are representative examples of the observed colony growth. Overexpression of EPHB6 in LIM2405 (**I**) and HCT15 (**J**) or EPHB6 knockdown in SW480 (**K**) and SW620 (**L**) did not affect their growth when injected subcutaneously in NOD/SCID immunodeficient mice. Par: parental cells; EV: empty vector cells; EPHB6: overexpressing cells; shNT: non-target shRNA control cells; shB6: EPHB6 knockdown cells.

Because EPH signaling has been shown to regulate cell adhesion and motility in several tumor types^21^, we used the *in vitro* systems generated to investigate whether EPHB6 regulates the motility of colon cancer cells. Using a wound healing assay we observed that modulation of EPHB6 levels did not alter the motility of colon cancer cells compared to the corresponding parental and control derivative lines (Fig. 2). In addition, because EPHB signaling through other family members can regulate cell proliferation in the normal intestinal epithelium and intestinal tumors^5^,^15^, we investigated whether EPHB6 regulates the growth of colon cancer cells. Overexpression of EPHB6 in LIM2405 and HCT15 cells or EPHB6 knockdown in SW480 and SW620 cells had no effect on the growth of these colon cancer cell lines on a solid substrate (Fig. 3A–D). Moreover, ectopic expression of EPHB6 in EPHB6-deficient colon cancer cells or downregulation of EPHB6 in cell lines with endogenous expression did not affect their anchorage-independent growth on a semi-solid substrate (Fig. 3E–H). In addition, although in breast cancer cells EPHB6 has been shown to regulate anoikis^22^, a type of programmed cell death induced upon cell detachment from a solid substrate, isogenic manipulation of EPHB6 levels did not affect the survival of colon cancer cells when grown under non-adherent condition (Supplementary Figure 2). Consistently, modulation of the levels of EPHB6 in colon cancer cells did not affect their growth when they were injected subcutaneously in NOD/SCID (nonobese diabetic/severe combined immunodeficiency) immunodeficient mice (Fig. 3I–L and Supplementary Figure 3) and no differences were observed in the proportion of proliferating cells (BrdU- or PCNA-positive) in the subcutaneous xenografts formed by cells with EPHB6 modulation compared to control cells (Supplementary Figure 4). As a control, EPHB6 overexpression/downregulation was confirmed by immunohistochemistry on subcutaneous tumor xenografts of these cells in immunodeficient mice (Supplementary Figure 5). Collectively, these results demonstrate that EPHB6 does not regulate the motility, anchorage dependence or proliferation of colon cancer cells.

### Investigation of the role of *EphB6* in intestinal tumorigenesis using a knockout mouse model

Although EPHB6 does not regulate the motility or growth of colon cancer cells *in vitro*, the loss of this EPH receptor could regulate tumor initiation and/or progression in the more complex context of the whole organism. Therefore, we used an *EphB6* knockout mouse model where part of exon 1 and the complete exon 2 and 3 were replaced by a PGK-neo cassette, resulting in the loss of EphB6 expression^23^. We found that *EphB6*^−/−^ animals were born at the expected Mendelian ratios (118/191/87 animals *EphB6* wild type, heterozygous and knockout; 29.8%, 48.2% and 22.0%, respectively; χ^2^ p = 0.28). Although other EPHB receptors have previously been shown to be important for cell sorting within the intestinal crypts^14^, inactivation of *EphB6* did not affect the distribution of enteroendocrine, goblet and Paneth cells in the small intestine compared to *EphB6* wild type animals (Supplementary Figure 6). Importantly, no changes in the incidence of spontaneous intestinal tumors were observed in *EphB6*^−/−^ mice at 21 months of age when compared to *EphB6*^+/+^ animals (Supplementary Figure 7), indicating that the loss of *EphB6* does not efficiently initiate tumor formation.

**Figure 4:**
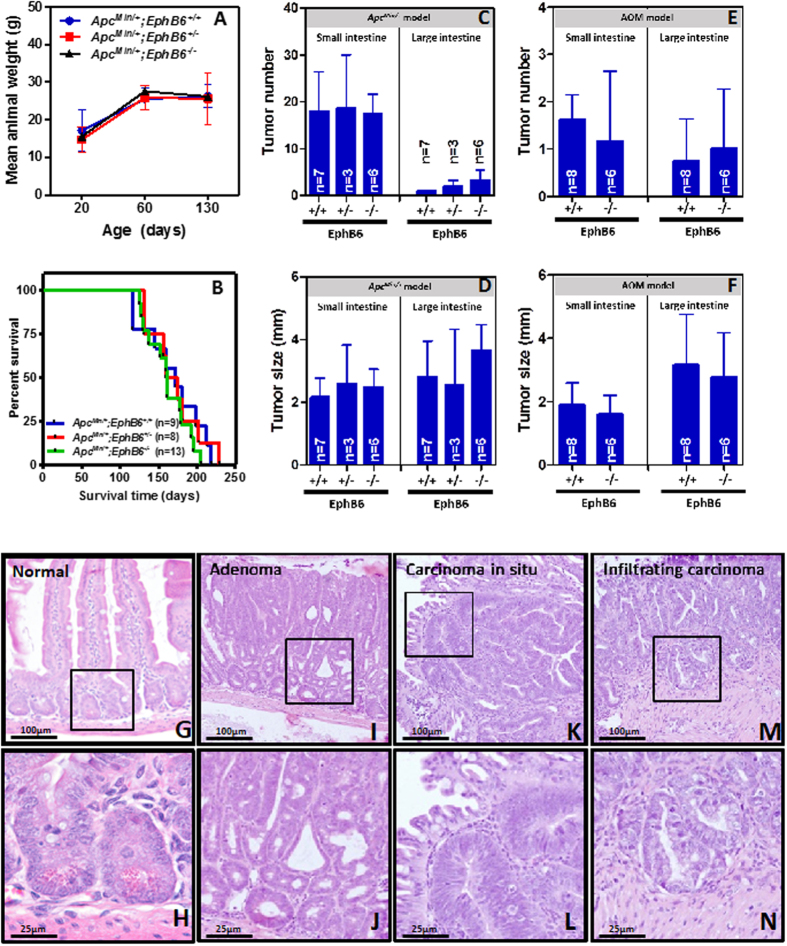
Inactivation of *EphB6* does not affect intestinal tumorigenesis in mouse models. (**A**) No differences were observed in the weight of animals bearing heterozygous *Apc* mutations and that were wild type, heterozygous or knockout for *EphB6* (Student’s t-test p > 0.6). (**B**) No differences were observed in the survival of *Apc*^*min*/+^ mice when either one or two copies of *EphB6* were inactivated (logrank test p > 0.27). (**C,D**) Number and size of the tumors observed in the small and large intestine of 19-week-old *Apc*^*min*/+^ mice that are *EphB6* wild type, heterozygous or knockout. The number of animals per group (n) is shown. (**E** and **F**) Number and size of intestinal tumors by *EphB6* genotype in the AOM model. (**G,H**) Representative micrographs of the normal small intestine stained with Hematoxylin and eosin at low and high magnification, respectively. Histology of a representative adenoma (**I,J**), carcinoma *in situ* (**K,L**) and infiltrating carcinoma (**M,N**).

**Figure 5:**
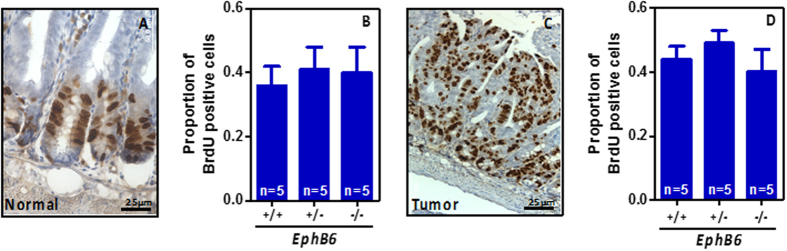
Proliferation in the normal intestinal epithelium and tumors. Panel (A) shows BrdU-positive cells in small intestinal crypts (brown staining). (**B**) Quantification of the proportion of BrdU-positive cells per intestinal crypt column. A minimum of 20 crypt columns were counted per animal. Panel (C) shows BrdU-positive cells in a representative small intestinal tumor, and quantification of the proportion of BrdU-positive cells is shown in panel (D). Five animals per group were scored. A minimum of 3 tumors per animal and at least 350 total cells per tumor were scored blinded from the animal ID. No significant differences were observed (Student’s t-test p > 0.05). N = number of animals.

Intestinal tumorigenesis was therefore initiated either genetically by crossing *EphB6* knockout animals with *Apc*^*min*/+^ mice carrying heterozygous mutations in the adenomatous polyposis coli (*Apc*) tumor suppressor gene^24^, or pharmacologically with the intestinal-specific carcinogen azoxymethane (AOM). Using the genetic model of intestinal tumor initiation we found no differences in the weight of *Apc*^*min*/+^ mice that were *EphB6*^+/+^, *EphB6*^+/−^ or *EphB6*^−/−^ at the age of 20, 60 or 130 days (two-way analysis of variance –ANOVA, p = 0.68; Fig. 4A). The lifespan of *Apc*^*min*/+^ mice was not affected by the loss of one or two copies of *EphB6* (Logrank test, p > 0.27; Fig. 4B). In good agreement with this finding, no differences between 19-week-old *Apc*^*min*/+^ mice that are either wild type, heterozygous or knockout for *EphB6* were observed in tumor number (Student’s t-test p > 0.18; Fig. 4C), tumors size (Student’s t-test p > 0.5; Fig. 4D) or the number of invasive adenocarcinomas (Student’s t-test p > 0.83; Supplementary Figure 8), in the small or large intestine. Representative examples of the different histological types of tumors observed in these animals are shown in Fig. 4G–N. Accordingly, no differences were observed in the number of proliferating cells in the normal small intestine or the tumors of *Apc*^*min*/+^; *EphB6*^+/+^, *Apc*^*min*/+^; *EphB6*^+/−^ and *Apc*^*min*/+^*; EphB6*^−/−^ mice (Fig. 5). Using an independent model of intestinal tumor initiation, we found that AOM treatment resulted in tumor formation both in the small and large intestine. However, consistent with the findings of the mouse model using genetic tumor initiation, no differences were observed in the number (Student’s t-test p > 0.49; Fig. 4E) or size (Student’s t-test p > 0.61; Fig. 4F) of the tumors in *EphB6* wild type and knockout mice after AOM treatment. Overall, these experiments indicate that, unlike the loss of other *EphB* receptors^5^,^6^, *EphB6* inactivation does not significantly contribute to intestinal tumor initiation or progression during the early stages of the tumorigenic process in murine models.

### The loss of EPHB6 contributes to colorectal cancer metastasis

**Figure 6:**
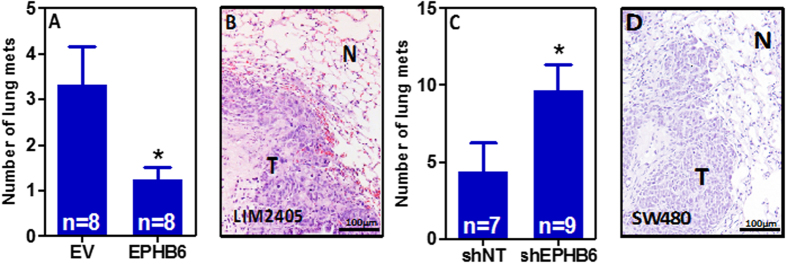
EPHB6 and lung metastasis. (**A,B**) Tail-vein injection of LIM2405 cells resulted in the formation of lung metastasis that were confirmed by Hematoxylin and eosin staining of histological sections of formalin-fixed, paraffin-embedded lungs (N: normal; T: tumor). A significant reduction in the number of lung metastasis was observed in the lungs of mice injected with isogenic EPHB6-overexpressing LIM2405 cells (LIM2405-EPHB6) compared to mice injected with the LIM2405-EV control cells. (**C,D**) EPHB6 knockdown in SW480 cells resulted in a significant increase in the number of metastatic lesions compared to control cells (non-target shRNA cells –shNT) in the lungs of NOD/SCID animals after tail-vein injection. The mean ± SEM is shown in panels (A) and (C). N: number of animals. Asterisks indicate statistically significant differences (Student’s t-test p < 0.05).

Because EPHB6 has previously been found to be important in the metastatic process in other tumor types^11^,^25^,^26^,^27^, we investigated the role of this EPH receptor in the metastatic spread of colon cancer cells. We injected LIM2405-EPHB6 or control LIM2405-EV cells in the tail-vein of NOD/SCID immunodeficient mice as an experimental model of metastasis. All animals were euthanized 37 days after cell injection and post-mortem dissection of their lungs revealed the presence of frequent metastasis (Fig. 6A–B). Reintroduction of EPHB6 into LIM2405 colon cancer cells resulted in a significant reduction in the number of lung metastases observed in NOD/SCID mice compared to animals injected with the control EPHB6-deficient cells (LIM2405-EV; Fig. 6A). Moreover, EPHB6 knockdown in SW480 colon cancer cells significantly increased the number of metastatic lesions observed in the lungs of NOD/SCID mice 52 days after tail-vein injection (Fig. 6C–D). When considered together, these results indicate that EPHB6 regulates the metastatic potential of colon cancer cells.

### EPHB6 expression in human colorectal tumors

**Figure 7:**
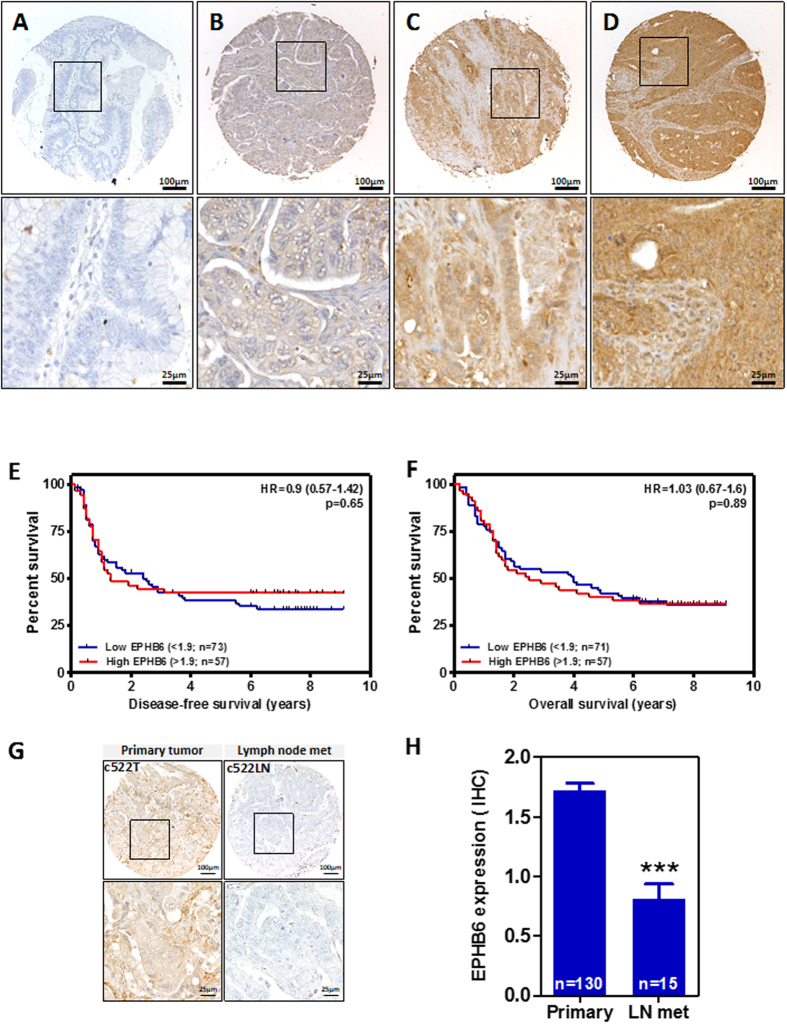
EPHB6 expression in primary colorectal tumors and patient survival. (**A,D**) The levels of EPHB6 protein expression were determined by immunohistochemistry using sections of a tissue microarray containing formalin-fixed, paraffin-embedded tumor samples from a cohort of 130 Dukes C colorectal cancer patients. Representative images of colorectal tumors with different levels of EPHB6 are shown in panels (**A,D**). No associations were observed between EPHB6 expression and disease-free (**E**) or overall (**F**) survival of these patients. The Logrank test p values are shown. (**G**) The levels of EPHB6 protein expression in a representative primary tumor and a matched lymph node metastasis is shown. (**H**) Histogram showing the average (±SEM) EPHB6 immunointensity score in primary colorectal tumors and lymph node metastasis (Student’s t-test p < 0.0001).

We next investigated whether the expression of EPHB6 was associated with the survival or other clinicopathological features of patients with colorectal cancer. EPHB6 expression levels were assessed by immunohistochemistry of sections of a tissue microarray containing formalin-fixed, paraffin-embedded (FFPE) tumor samples from a cohort of 130 colorectal cancer patients with locally advanced disease (Dukes C; Supplementary Table 1). The specificity of the antibody used was confirmed on FFPE samples of subcutaneous tumor xenografts of the engineered isogenic cell line systems in immunodeficient mice (Supplementary Fig. 5). Significant variability was observed in the levels of expression of EPHB6 in colorectal tumors (Fig. 7A–D). However, EPHB6 expression was not associated with the overall or disease-free survival of colorectal cancer patients (Fig. 7E–F) irrespectively of the cutoff value used to define the high and low EPHB6 groups (Supplementary Fig. 9). In addition, no associations were found between EPHB6 expression levels and other clinicopathological features of these patients including patient sex, age, or tumor site (colon/rectum), the administration of adjuvant treatment, as well as some molecular characteristics of the tumors, including microsatellite instability, allelic loss of chromosome 18q and the presence of mutations in *TP53* or *KRAS* (Supplementary Table 1).

However, significantly lower levels of EPHB6 expression were observed in lymph node metastasis compared to primary colorectal tumors (Fig. 7G–H). These results are in good agreement with the role of EPHB6 in the metastatic potential of colon cancer cells observed using the experimental model of metastasis in immunocompromised NOD/SCID mice, and further indicate that the loss of EPHB6 contributes to the metastatic spread of colon cancer cells.

## Discussion

Previous investigations have established the important role of EPH signaling during the oncogenic process in different human organs, including the gastrointestinal tracts^1^,^2^,^3^,^4^,^5^,^6^. Despite the lack of kinase activity, EPHB6 has been shown to have tumor suppressor activity in different tumor types, such as lung^13^,^27^ and breast^11^,^20^ cancer. Although the loss of EPHB2, EPHB3 and EPHB4 has been shown to significantly contribute to colorectal cancer progression^5^,^6^,^28^, the role of EPHB6 in colorectal cancer has not been investigated. In this study we used isogenic *in vitro* systems, animal models and large collections of primary colorectal tumor samples to investigate the functional relevance of EPHB6 during colorectal tumorigenesis.

EPH signaling regulates cell motility/migration in different tumor types, including colorectal cancer^5^,^6^,^29^,^30^. Moreover, EPHB6 has been shown to regulate the motility and invasive potential of other tumor types, including breast^11^ and lung^13^ tumors. However, we found here that the motility of colon cancer cells was not affected by the modulation of EPHB6 levels in four independent tumor cell lines, indicating that this function of EPHB6 in cancer cells is context-dependent. This is consistent with the observation that other type B EPH receptors such as EPHB4, have opposite effects on cell motility/invasion in different tumor types^5^,^31^.

In addition, EPHB signaling has been shown to be an important regulator of cell proliferation in both the normal intestinal epithelium and intestinal tumors^5^,^6^,^15^. Therefore, we investigated the role of EPHB6 in this process, using both *in vitro* systems and animal models. We found that EPHB6 does not regulate the growth of colon cancer cells, either on a solid substrate, a semisolid substrate or when grown as subcutaneous xenografts in immunodeficient mice. Moreover, in mice with targeted inactivation of endogenous *EphB6*, no differences in proliferation were observed in either the normal intestinal epithelium or intestinal tumors initiated by *Apc* mutations. Collectively, these results demonstrate that EPHB6 does not regulate the proliferation of intestinal epithelial cells either in the normal mucosa or in primary intestinal tumors. Using animal models, we and others have previously shown that inactivation of other EphB receptors, such as EphB2, EphB3 or EphB4, is an important step in the early stages of the intestinal tumorigenic process^5^,^6^,^15^. Here we demonstrate that, although EPHB6 germline mutations have been suggested to predispose to colorectal cancer^32^, the loss of EphB6 does not significantly contribute to intestinal tumor initiation, or the progression of tumors initiated either genetically (*Apc* mutations) or by carcinogen exposure (AOM) in animal models.

Colorectal cancer mortality is largely due to the metastatic spread of the disease to distant organs and the molecular mechanisms regulating this complex, multistage process are not fully understood. However, the mouse models used here develop mostly benign adenomas and are not ideally suited for investigating the role of EPHB6 in the later stages of tumor development. Although increased cell motility is often associated with enhanced metastatic potential, *in vitro* assays cannot fully capture the complexity of the multistage metastatic process. Therefore, an experimental animal model of metastasis was used to directly investigate the possible role of EPHB6 in the late stages of metastatic spread of colon cancer cells. Importantly, we found that the reintroduction of EPHB6 into EPHB6-deficient cells significantly reduced the metastatic growth of colon cancer cells in the lungs of immunocompromised NOD/SCID mice after tail-vein injection. Conversely, EPHB6 knockdown resulted in an increased number of metastatic lesions in this experimental model of metastasis. Moreover, reduced EPHB6 expression was observed in lymph node metastases compared to primary tumors of patients with locally advanced colorectal cancer. Collectively, these results demonstrate for the first time a role for EPHB6 in the metastatic spread of colorectal tumors. Although these findings await further validation using additional experimental approaches, the effects of EPHB6 modulation on the metastatic potential of colon cancer cells are consistent with the role of this EPH receptor in the metastatic progression of other tumor types, such as melanoma, breast and lung tumors^11^,^13^,^25^,^26^,^27^,^33^.

Reduced EPHB6 expression has been reported to be associated with poor prognosis of patients with different types of cancer, including melanoma and neuroblastoma^26^,^34^. Interestingly, although low tumor levels of EPHB6 protein in a series of 79 patients with colorectal cancer of Dukes stage A–D were recently reported to be associated with reduced patient survival^35^, no associations were observed here between EPHB6 expression and survival of a cohort of 130 patients with Dukes C colorectal cancer. This apparent discrepancy may be due to the different antibodies used in these two studies for the assessment of EPHB6 levels in tumor samples. Moreover, Peng *et al* found that reduced EPHB6 levels are associated with advanced disease stage^35^, suggesting that the levels of EPHB6 may not be an independent prognostic factor for patients with colorectal cancer. This would be consistent with the lack of prognostic value observed here for EPHB6 tumor levels in a stage-specific cohort of colorectal cancer patients.

Despite being a kinase-dead receptor, EPHB6 can form hetero-receptor complexes with other EPHB receptors such as EPHB1 and EPHB4 and undergoes transphosphorylation upon ligand binding^9^,^20^. Downstream signaling regulating cell adhesion and invasion in breast cancer cells involves the CBL-dependent phosphorylation of ABL^20^. Although we have previously shown that EPHB4 has tumor suppressor activity in colorectal tumors^3^,^5^, it is currently not clear to what extent the interaction between EPHB4 and EPHB6 is necessary for tumor/metastasis suppression by these EPH receptors, and the detailed downstream signaling mechanisms remain to be elucidated.

In summary, using an *EphB6* mouse knockout model we found that the loss of EphB6 does not initiate intestinal tumorigenesis and is not involved in the early tumor progression through the adenoma-to-carcinoma transition. Moreover, EPHB6 does not regulate the growth of tumor cells *in vitro* or *in vivo* either in a xenograft model or in mouse knockout models after genetic/carcinogen tumor initiation. However, we report here for the first time that the loss of EPHB6 contributes to the metastatic spread of colorectal tumors.

## Materials and Methods

### Cell culture

All the colorectal cancer cell lines used in this study were maintained in Dulbecco’s modified Eagle’s medium (DMEM; PAA Laboratories) supplemented with 10% fetal bovine serum (PAA Laboratories) and 1x antibiotic-antimycotic (10,000 U penicillin, 10,000 μg streptomycin and 25 μg/ml amphotericin B; Invitrogen) at 37 °C in a humidified atmosphere with 5% CO_2_. All cell lines were obtained from the American Type Culture Collection (ATCC) and cell line identity was validated by Affymetrix SNP6.0 array analysis. To stably overexpress EPHB6, LIM2405 and HCT15 cell lines were transfected with a construct expressing N-terminally Hemagglutinin (HA) tagged EPHB6 or the corresponding empty vector control (pDISPLAY Vector, Invitrogen) using Lipofectamine 2000 (Invitrogen). After neomycin selection (500 μg/ml), cells were stained with an anti-HA antibody (clone 12CA5; Roche) and HA-positive cells sorted with a Cell Sorter FACSAria (Becton Dickinson). After expanding the HA-positive population, the sorting was repeated. SW480 and SW620 cells were transduced with a lentiviral vector (pLKO; Sigma) expressing a shRNA with confirmed specificity to EPHB6 (Sequence: CCG GAT GTG GGA AGT GAT GAG TTA TCT CGA GAT AAC TCA TCA CTT CCC ACA TTT TTT; TRCN0000010677, Sigma).

### Clinical samples

Samples from colorectal cancer patients with locally advanced disease (Dukes C) were collected at nine hospitals from Finland (EKKS, HYKS, KAKS, KOKS, KSKS, KYS, MKS, PKKS and SKS) and informed consent for genetic analysis of the tumor sample was provided by each patient as previously described^36^,^37^. All methods were carried out in accordance with relevant guidelines and regulations. All experimental protocols were approved by the appropriate ethics review committees as described^36^,^37^. The mean follow up of these patients was 7.3 years (range from 3.1 to 9.5 years). For tissue microarray preparation, areas containing a high proportion of tumor cells were selected after histological examination of hematoxylin and eosin stained tumor sections. As previously described^3^,^38^, triplicate 0.6 mm cores from every sample were arrayed in a fresh paraffin block using a Beecher Instrument tissue arrayer (Silver Spring, MD). Unstained 4 μm sections from the tissue microarray were mounted on slides coated with 3-aminopropyl-triethoxy-silane (Sigma, St Louis, MO).

### Western blotting

Cell cultures were harvested at 70% confluence and cell pellets resuspended in radioimmunoprecipitation (RIPA) assay buffer (0.1% SDS, 1% NP40 and 0.5% Na-deoxycholate in PBS) complemented with protease inhibitors (Pepstatine 5 μg/μl, PMSF 0.3 mM, Aprotinine 1 μg/μl and Sodium orthovanadate 100 μM). Aliquots of total protein (100 μg) were loaded on a 10% acrylamide gel. After gel electrophoresis, proteins were transferred to a PVDF membrane and probed with anti-EPHB6 (1:500, SAB4503476, Sigma), anti-Actin (1:1000; Sigma) or anti-Tubulin (1:1000; clone H-300; Santa Cruz) as previously described^5^.

### RNA extraction and quantitative RT-PCR

Cell cultures were harvested at 70% confluence and total RNA was extracted using TRI Reagent (Molecular Research Center) according to the manufacturer’s instructions. Total RNA (500 ng) was reverse transcribed using the High Capacity cDNA Reverse Transcription kit (Applied Biosystems), and relative EPHB6 mRNA levels were assessed by Real-Time PCR using SYBR Green Master Mix (Applied Biosystems, Branchburg, NJ). We used 18 S rRNA (Taqman Master Mix) as a standardization control for the 2^−ΔΔCt^ method as described before^39^. The primers used were EPHB6-qPCR-F: 5′-CTGGAGTGCTACCAGGACAA-3′; EPHB6-qPCR-R: 5′-GGCAGGTCTTCTAGGCTGAG-3′; 18 S rRNA-F: 5′-AGTCCCTGCCCTTTGTACACA-3′; 18 S rRNA-R: 5′-GATCCGAGGGCCTCACTAAAC-3′; 18 S Probe: 5′-FAM-CGCCCGTCGCTACCGATTGG-TAMRA-3′.

### Anchorage-independent growth assay

2.5 × 10^4^ cells were cultured in complete DMEM medium containing 0.3% noble agar (USB Corporation) and plated on top of 0.6% noble agar in DMEM medium. Cultures were maintained at 37 °C in a 5% CO_2_ incubator for 2–3 weeks. Colonies were stained with nitrotetrazolium blue chloride (1 mg/ml; Sigma) and macroscopically visible colonies were scored. Experiments were carried out three times in triplicate.

### Wound-healing assay

Cells were cultured in 6-well plates and allowed to grow to 90% confluence. The cell monolayer was scratched with a sterile micropipette tip and the wound region was allowed to heal by cell migration. The area that remained clear of cells after 0, 12, 24 and 48 h was quantified with ImageJ (National Institutes of Health, NIH) and compared with the area of the wound at time zero. Experiments were run three times in triplicate.

### Cell Growth quantification

A sulforhodamine B (SRB) assay was used for cell growth determination. Cells were seeded in a 96-well plate at a density of 2000 cells/well. Cells were fixed by adding 50 μl of 50% trichloroacetic acid after 24, 48, 72, 96, 120 and 144 h of culture. Cells were then washed with water and air-dried. Plates were stained with 0.4% SRB in 1% acetic acid for 30 min, rinsed three times with 1% acetic acid and air-dried. SRB was solubilized in 200 μl/well of 10 mM Tris pH 10, and the absorbance at 590 nm recorded using a Benchmark Plus microplate spectrophotometer (BioRad Laboratories, Hercules, CA). Measurements were adjusted for background and the absorbance of the corresponding time 0 plates was subtracted. Experiments were run three times with eight replicates per experiment.

### Anoikis induction and flow cytometry

Tissue culture plates were coated with poly(2-hydroxyethyl methacrylate) (poly-HEMA; Santa Cruz) by adding 1 ml of a 20 mg/ml poly-HEMA/96% ethanol solution to each well of a 6-well plate. After drying the plates for 1 h, cells (5 × 10^5^) were seeded and allowed to grow for 72 h in complete DMEM medium (10% FBS and 1x antibiotic antimycotic). Cells were then harvested, washed with PBS and resuspended in 1 ml of propidium iodide buffer (50 μg/ml propidium iodide, 0.1% sodium citrate, and 0.1% Triton X-100). Cells were stained for 1 h at 4 °C, and 10,000 cells were analyzed for DNA content using a FacsCalibur Flow Cytometer (Becton Dickinson). The percentage of cells with a subdiploid DNA content was quantified using FlowJo software (Treestar, Inc., San Carlos, CA).

### Xenograft model

NOD/SCID mice (NOD.CB17-Prkdc^scid^/NCrCrl, Charles River) 8 weeks old were injected subcutaneously with LIM2405-EPHB6-HA cells (1.5 × 10^6^; n = 6 mice), HCT15-EPHB6-HA cells (5 × 10^6^; n = 6 mice), SW480-shEPHB6 cells (5 × 10^6^; n = 8 mice) or SW620-shEPHB6 cells (2 × 10^6^; n = 9 mice) in the right flank and with the same number of the corresponding matched control lines in the left flank. Tumor size was measured using a caliper three times per week. Tumor volume (V) was calculated with the formula: *V* = (*L* × *W*^2^) × 0.52, where *L* is the length and *W* is the width of a xenograft. All animal experiments were carried out under protocols approved by the Vall d’Hebron Ethical Committee for Animal Experimentation and the appropriate governmental agency and carried out in accordance with the approved guidelines.

### Mouse knockout strains and azoxymethane treatment

The C57BL/6J-Apc^min^/J strain was obtained from The Jackson Laboratory (stock number 002020). These mice carry a heterozygous mutation in *Apc*. The *EphB6* knockout mouse has been previously described^23^ and is on a C57BL/6 genetic background. Part of exon 1 and exons 2 and 3 were replaced by a PGK-neo cassette. Male *Apc*^*min*/+^; *EphB6*^+/+^ mice were crossed with female *Apc*^+/+^*; EphB6*^−/−^ mice to obtain *Apc*^*min*/+^; *EphB6*^+/−^ males and *Apc*^+/+^*; EphB6*^+/−^ females that were subsequently crossed to obtain the *Apc*^*min*/+^*; EphB6*^+/+^, *Apc*^*min*/+^; *EphB6*^+/−^ and *Apc*^*min*/+^; *EphB6*^−/−^. For animal ‘survival’ curves, the wellbeing of the mice was check three times per week and animals were euthanized by cervical dislocation when they became lethargic. Eleven-week-old *EphB6*^+/+^ and *EphB6*^−/−^ (both *Apc*^+/+^) mice were i.p. injected with the intestine-specific carcinogen azoxymethane (AOM; 10 mg/kg; Sigma) weekly for 9 weeks and sacrificed 53 weeks after the last AOM injection. All animals were i.p. injected with 100 mg/kg bromodeoxyuridine (BrdU) 2 h before being euthanized. Twenty-one-month-old mice (*EphB6*^+/+^ and *EphB6*^−/−^), 17-month-old AOM treated mice (*EphB6*^+/+^ and *EphB6*^−/−^) or 19-week-old *Apc*^*min*/+^ mice (*Apc*^*min*/+^; *EphB6*^+/+^*, Apc*^*min*/+^; *EphB6*^+/−^ and *Apc*^*min*/+^ *EphB6*^−/−^) were euthanized by cervical dislocation, the small and large intestines were dissected, measured, weighed and opened longitudinally. Tumor size and number were scored under a dissecting microscope. The intestine was rolled longitudinally using the ‘Swiss roll’ technique^40^ with the mucosa side inwards and the distal part of the intestine toward the center of the roll, then fixed with 10% formalin and embedded in paraffin.

### Experimental metastasis model

LIM2405-EPHB6 (1.5 × 10^6^; n = 8), control LIM2405-EV cells (1.5 × 10^6^; n = 8), SW480-shEPHB6 (4 × 10^6^; n = 9) and control SW480-shNT (4 × 10^6^; n = 7) cells suspended in 100 μl PBS were injected through the tail-vein of 7–8-week-old NOD/SCID mice using 25-gauge needles. All animals were i.p. injected with 100 mg/kg BrdU 2 h before being euthanized 37 days or 52 days post injection for the LIM2405 cells and the SW480 cells, respectively. The lungs were fixed with 4% paraformaldehyde and included in paraffin.

### Histology and immunohistochemistry

Sections from formalin-fixed, paraffin-embedded tissues (4 μm) were immunostained with NovoLink polymer detection system (Novocastra Laboratories) according to manufacturer’s instructions after antigen retrieval with 10 mM citrate buffer pH 6.0 for 20 min at 120 °C in autoclave. The sections were incubated with mouse monoclonal anti-BrdU (1/15 dilution of the hybridoma supernatant; Developmental Studies Hybridoma Bank) or mouse monoclonal anti-EPHB6 (1/200 dilution; Abnova; clone 5D8) primary antibodies at 4 °C, overnight. Anti-EPHB6 immunostaining intensity was determined using a semiquantitative scale ranging from 0 (no staining) to 3 (highest intensity) blinded from the clinical data. Associations between EPHB6 levels and patient survival were assessed using the Cutoff Finder R Package^41^. Antigen retrieval for lysozyme IHC (N1515; Dako; ready to use) was done in 100 °C 1 mM EDTA (10 min). Biotinilated anti-PCNA (1/6000; clone PC10, Biolegend) and peroxidase-conjugated streptavidin (1/2000 dilution; Spring Bioscience) were used for immunodetection of PCNA. Slides were counterstained with Mayer’s Hematoxylin dehydrated and mounted with DPX mounting medium (Panreac Quimica). The total number of BrdU-positive cells was scored blinded from the animal ID. Goblet cell staining was done with 1% alcian blue (Sigma) in 3% acetic acid for 30 min and counterstained with Hematoxylin. Enteroendocrine cells were stained using Grimelius silver staining as described^42^. Briefly, sections were dewaxed, rehydrated, post-fixed with Bouin’s solution (Sigma) for 1 h at 37 °C, rinsed once in 70% Ethanol, twice in distilled water and immersed in silver solution (0.5% silver nitrate in 0.1 M acetate buffer pH 5.8) for 3–4 h at 60 °C. Then, sections were immersed in Bodian solution (5% anhydrous sodium sulfate and 1% hydroquinone in water) for 5 min at 60 °C, fixed in 2% sodium thiosulphate, washed in PBS, dehydrated and mounted. Part of the histological processing was performed by the ICTS “NANBIOSIS”, (Unit 20; CIBER-BBN; Vall d’Hebron Institute of Research).

## Additional Information

**How to cite this article**: Mateo-Lozano, S. *et al*. Loss of the EPH receptor B6 contributes to colorectal cancer metastasis. *Sci. Rep.* **7**, 43702; doi: 10.1038/srep43702 (2017).

**Publisher's note:** Springer Nature remains neutral with regard to jurisdictional claims in published maps and institutional affiliations.

## Supplementary Material

Supplementary Materials
