# Supplementary material for: Loss of the EPH receptor B6 contributes to colorectal cancer metastasis
Source: Sci Rep. 2017 Mar 6;7:43702. doi: 10.1038/srep43702 (PMC5337985; doi:10.1038/srep43702)

SUPPLEMENTARY MATERIALS

Loss of the EPH receptor B6 contributes to colorectal cancer metastasis

Silvia Mateo-Lozano[1], Sarah Bazzocco[1], Paulo Rodrigues[1], Rocco Mazzolini[1], Elena Andretta[1], Higinio Dopeso[1], Yolanda Fernández[2,3,4], Edgar del Llano[1], Josipa Bilic[1], Lucía Suarez-López[2,3], Irati Macaya[1], Fernando Cartón-García[1], Rocio Nieto[1], Lizbeth Jimenez[1], Priscila Marcondes[1], Yaiza Nuñez[1], Elsa Afonso[1], Karina Cacci[5], Javier Hernández-Losa[5], Stefania Landolfi[5], Ibane Abasolo[2,3], Santiago Ramón y Cajal[5], John M. Mariadason[6], Simo Schwartz Jr[2,3], Toshimitsu Matsui[7], Diego Arango[1*].

Supplementary Figure 1

A

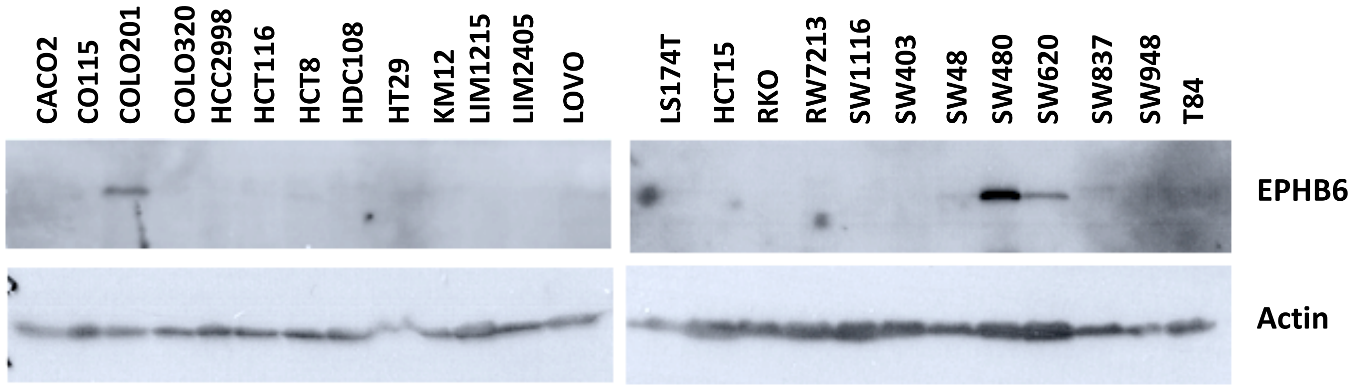

B

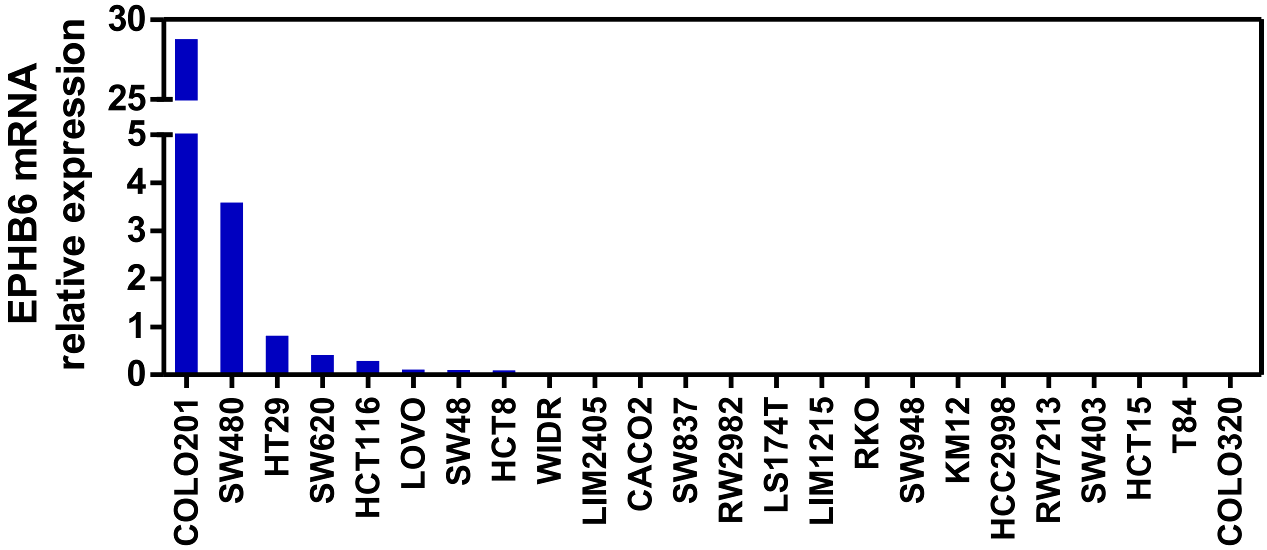

Supplementary Figure 1: EPHB6 levels in colorectal cancer cell lines. A) The relative protein levels of EPHB6 were assessed by Western blotting in a panel of 25 colorectal cancer cell lines. B) The relative mRNA levels of EPHB6 were assessed by quantitative Real-Time RT-PCR in 24 of these colorectal cancer cell lines.

Supplementary Figure 2

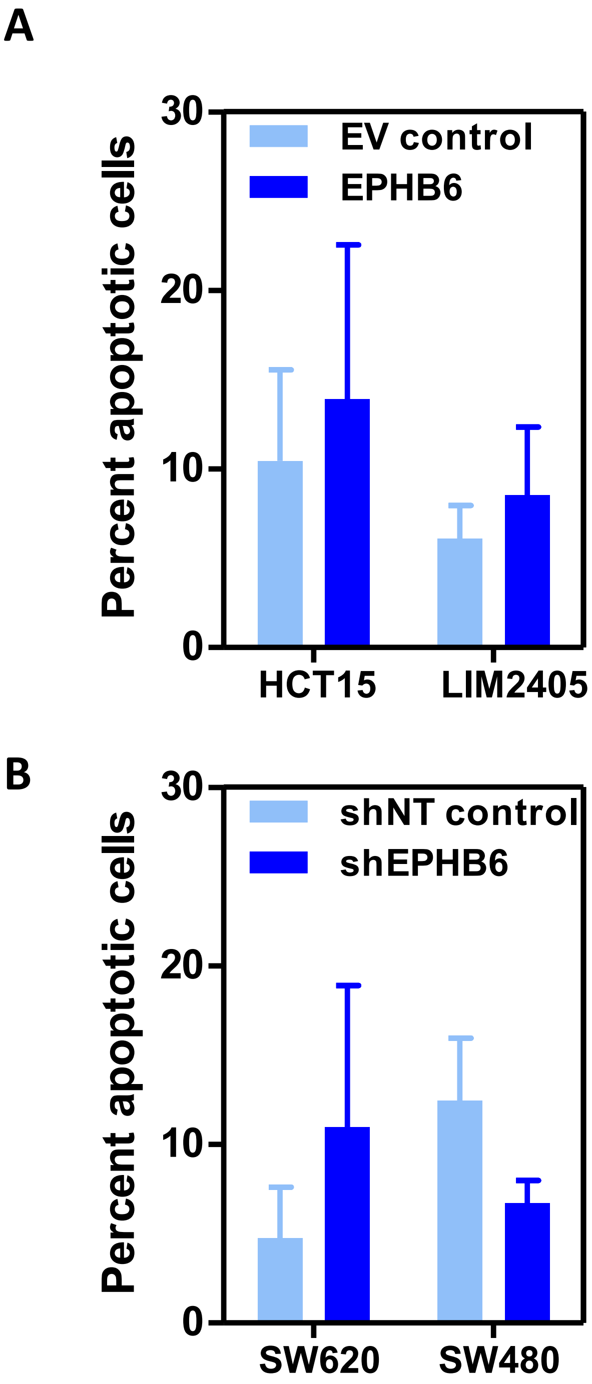

Supplementary Figure 2: Effects of EPHB6 modulation on anoikis. The number of apoptotic cells after growth in non-adherent conditions was quantified by propidium iodide staining and FACS analysis in colon cancer cells with EPHB6 overexpression (**A**) or downregulation (**B**). The mean ± SEM of three independent experiments carried out in triplicate is shown.

Supplementary Figure 3

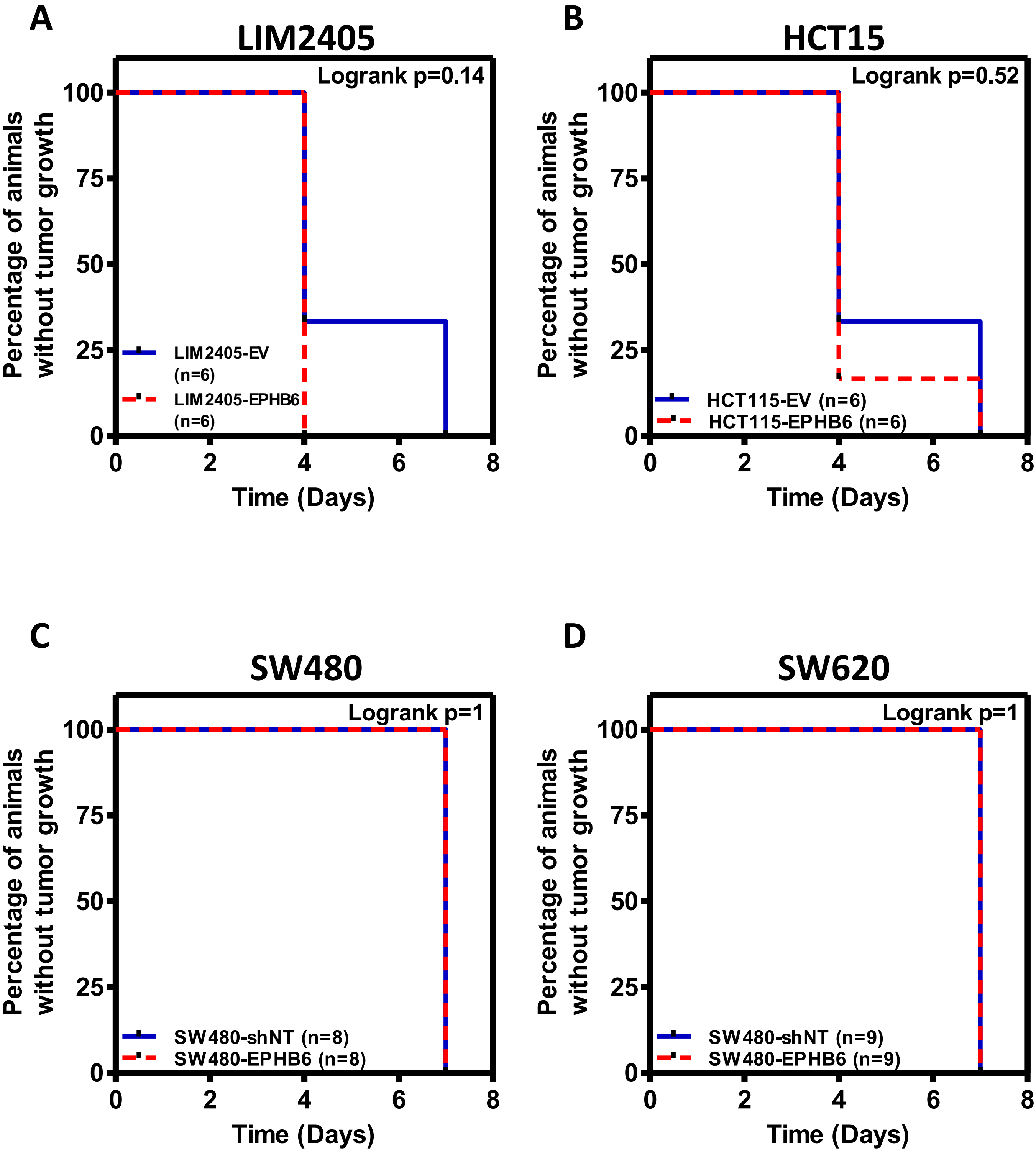

Supplementary Figure 3: Tumor latency after subcutaneous implantation of colon cancer cells. Kaplan-Meier curves showing differences in the time to initial tumor detection after subcutaneous implantation in NOD/SCID mice of EPHB6 overexpressing LIM2405 (A) and HCT15 (B) cells or EPHB6 knockdown SW480 (C) and SW620 (D) cells, compared to the corresponding control cells. n=number of animals. Logrank p>0.14.

Supplementary Figure 4

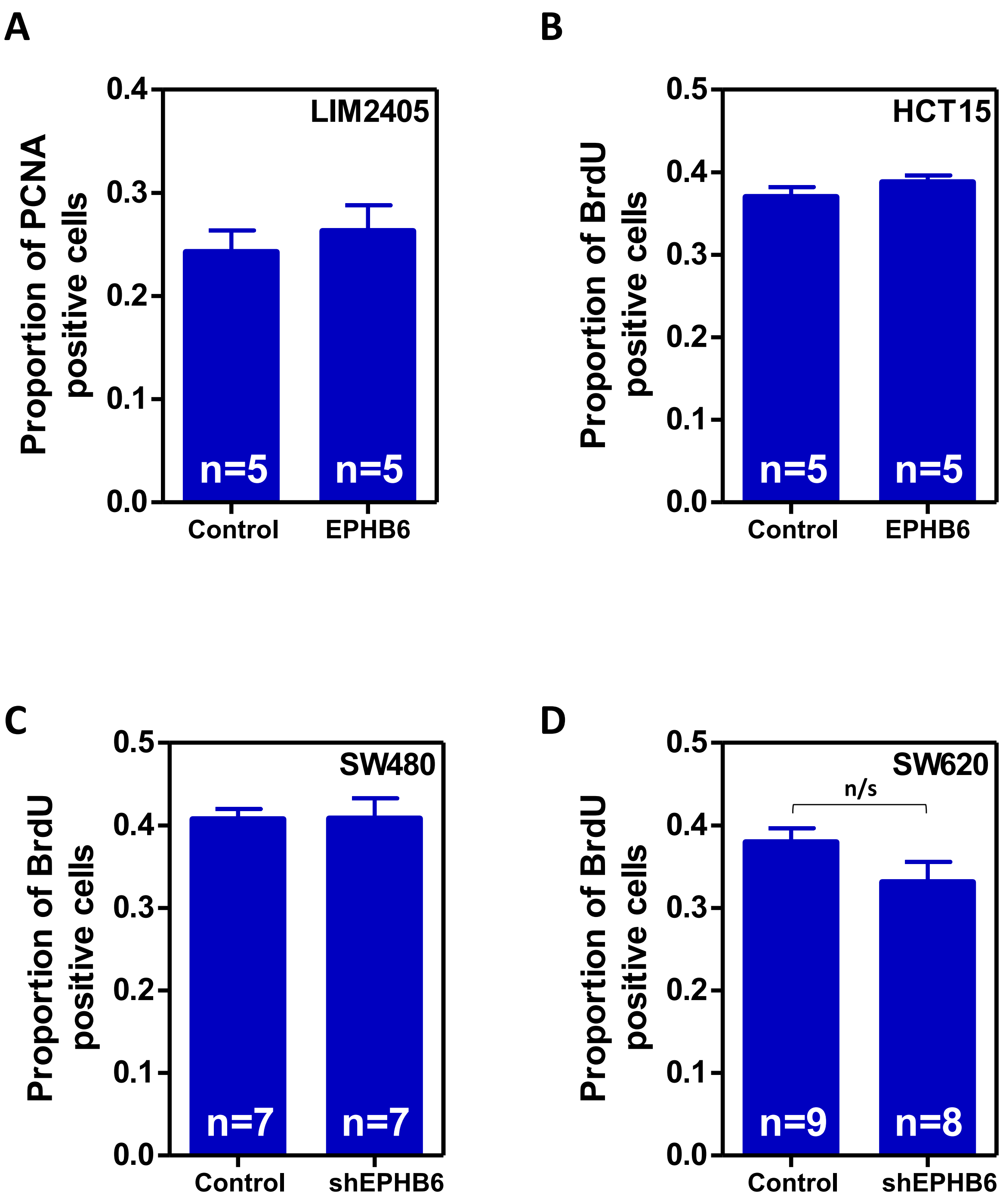

Supplementary Figure 4: Proliferation in subcutaneous xenografts of colon cancer cells with modulation of EPHB6 expression. The number of proliferating cells after EPHB6 overexpression in LIM2405 (A) and HCT15 (B) cells or EPHB6 knockdown in SW480 (C) and SW620 (D) cells was quantification by scoring the number of PCNA (A) or bromodeoxyuridine (BrdU; B-D) immunostained cells in subcutaneous xenografts of these lines in NOD/SCID mice. The mean ± SEM is shown. n/s: non significant p value ($p>0.05$; Student's T-Test).

Supplementary Figure 5

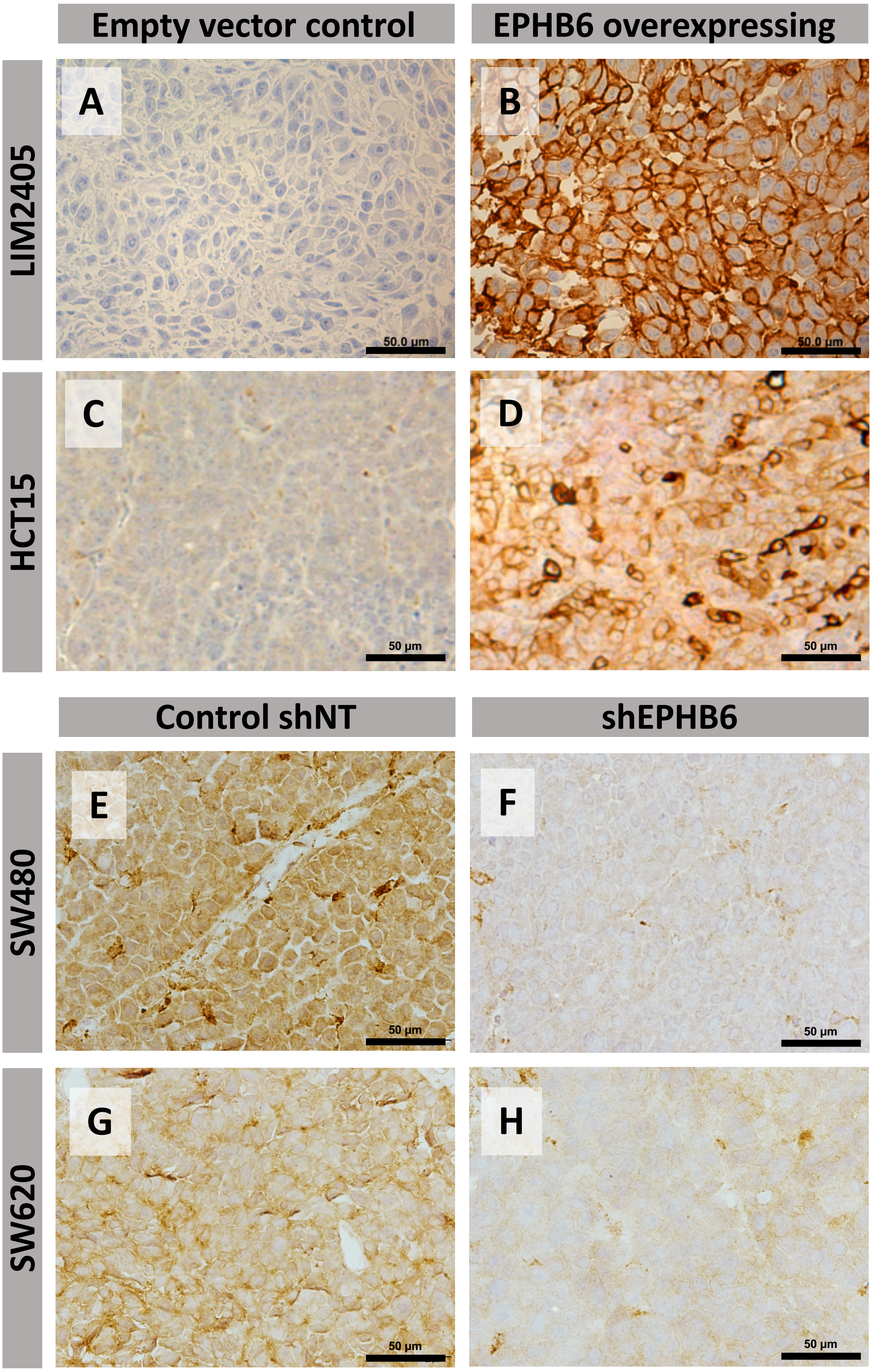

Supplementary Figure 5: Confirmation of EPHB6 modulation in subcutaneous xenografts. Immunohistochemistry with a mouse monoclonal anti-EPHB6 antibody (Abnova; clone 5D8) demonstrated EPHB6 overexpression on LIM2405 (A-B) and HCT15 (C-D) subcutaneous xenografts. Reduced EHPB6 levels were observed in the subcutaneous xenografts of SW480-shEPHB6 (F) and SW620-shEPHB6 (H) cells compared to the corresponding SW480-shNT (E) and SW620-shNT (H) control cells.

Supplementary Figure 6

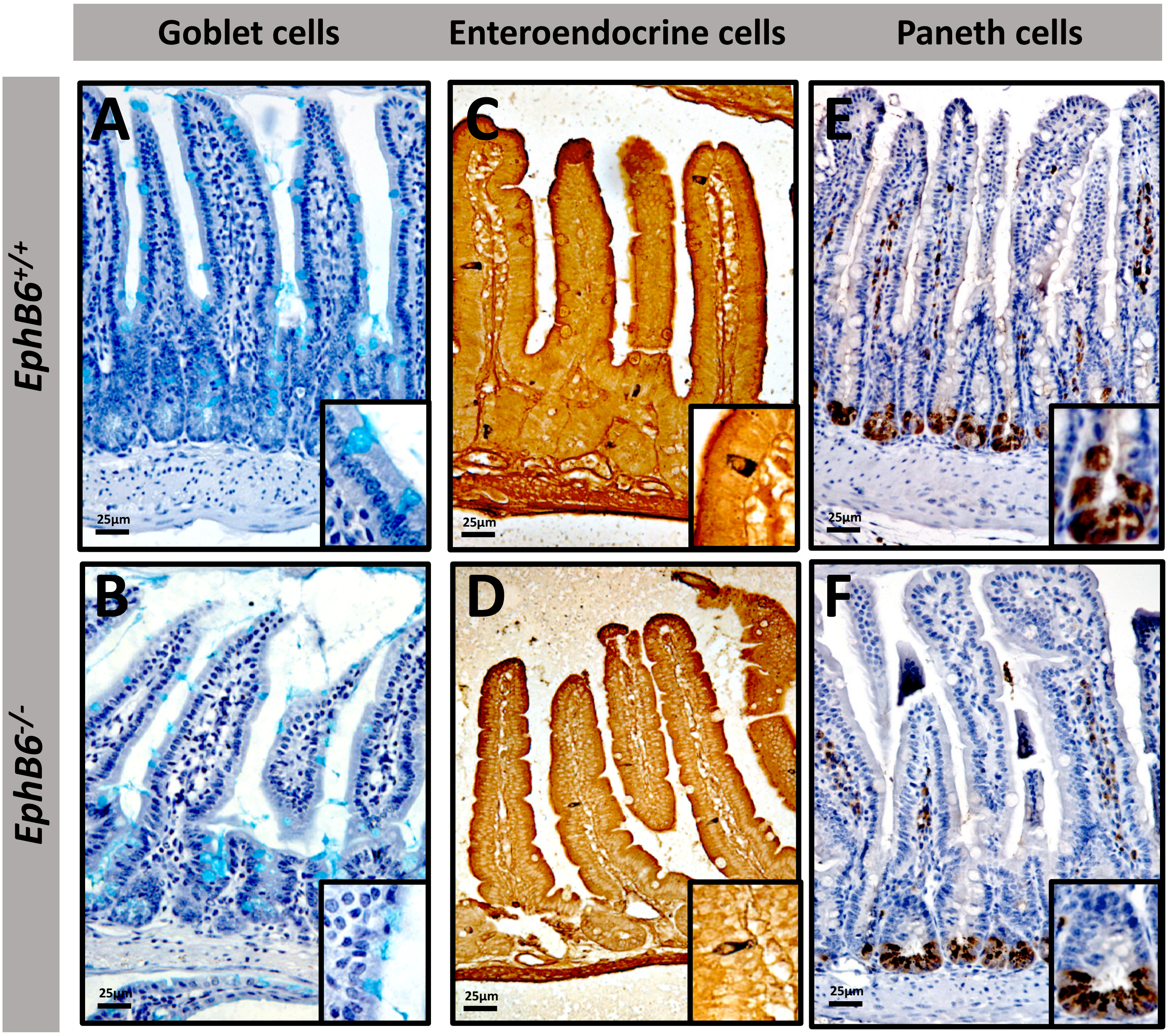

Supplementary Figure 6: No defects on epithelial cell positioning are observed in *Ephb6* knockout mice. No gross abnormalities were observed in the localization of goblet (A-B), enteroendocrine (C-D) or Paneth cells (E-F) in the small intestine of *EphB6* wild type (A, C and E) or knockout (B, D and F) mice. Cells with positive staining are highlighted in the figure insets.

Supplementary Figure 7

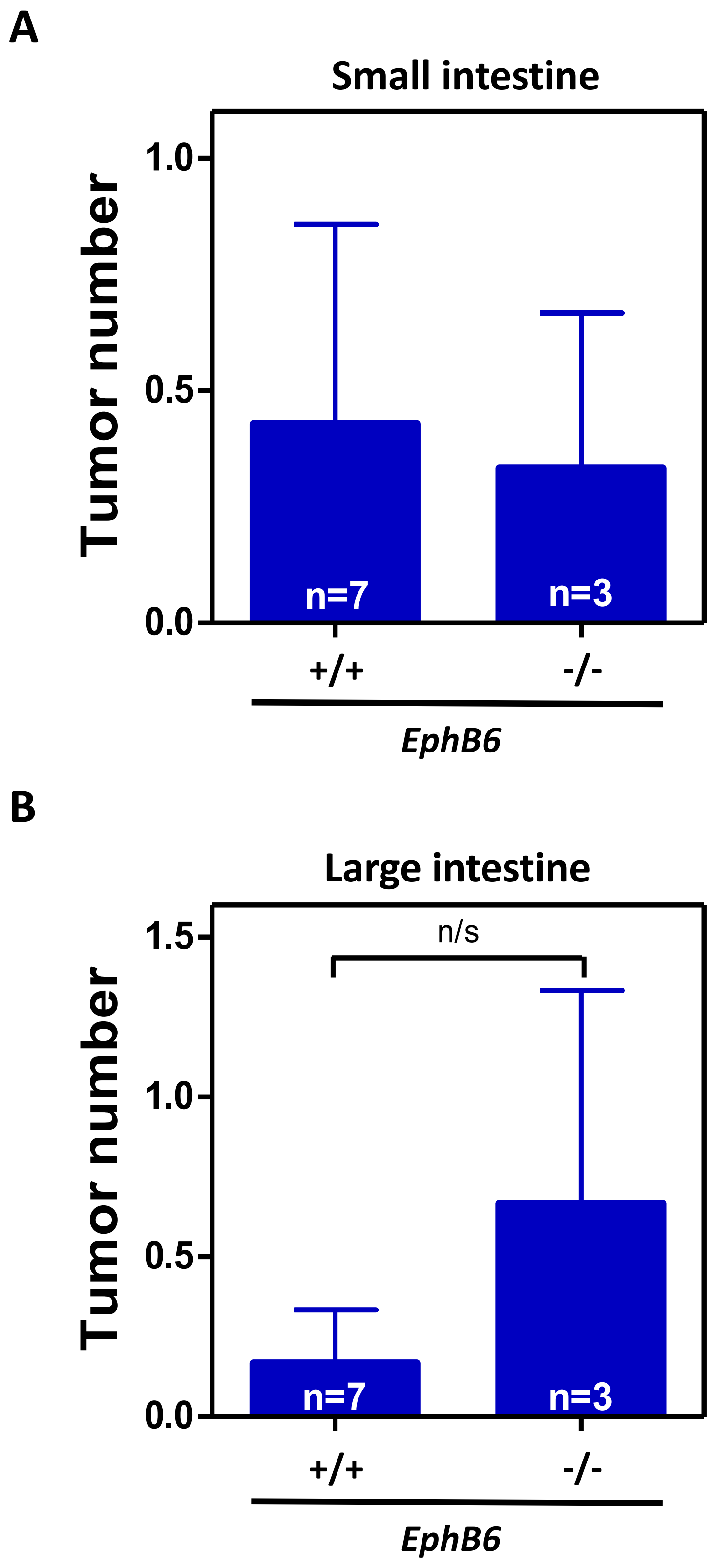

Supplementary Figure 7: Number of tumors in *EphB6* knockout mice. The number of tumors in *EphB6* wild type and knockout mice was assessed at 21 months of age in the small (A) and large (B) intestine. The number of animals per group (n) is shown. The mean ± SEM is shown. n/s: non significant p value ($p>0.05$; Student's T-Test).

Supplementary Figure 8

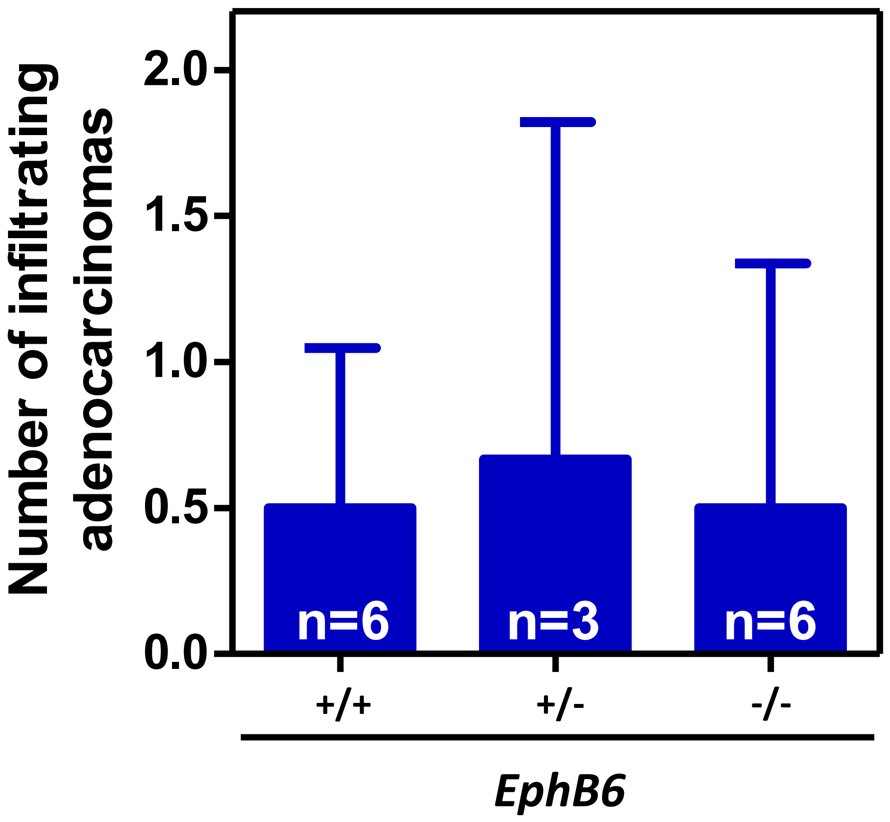

Supplementary Figure 8: Number of invasive adenocarcinomas. The number of infiltrating carcinomas was determined on histological sections of formalin-fixed, paraffin-embedded small intestines by an experienced pathologist blinded from the animal ID. No significant differences were observed in the number of infiltrating carcinomas in the small intestine of *Apc*$^{min/+}$ animals that were *EphB6* wild type, heterozygous or knockout. The mean ± SEM is shown. n: number of animals.

Supplementary Figure 9

A

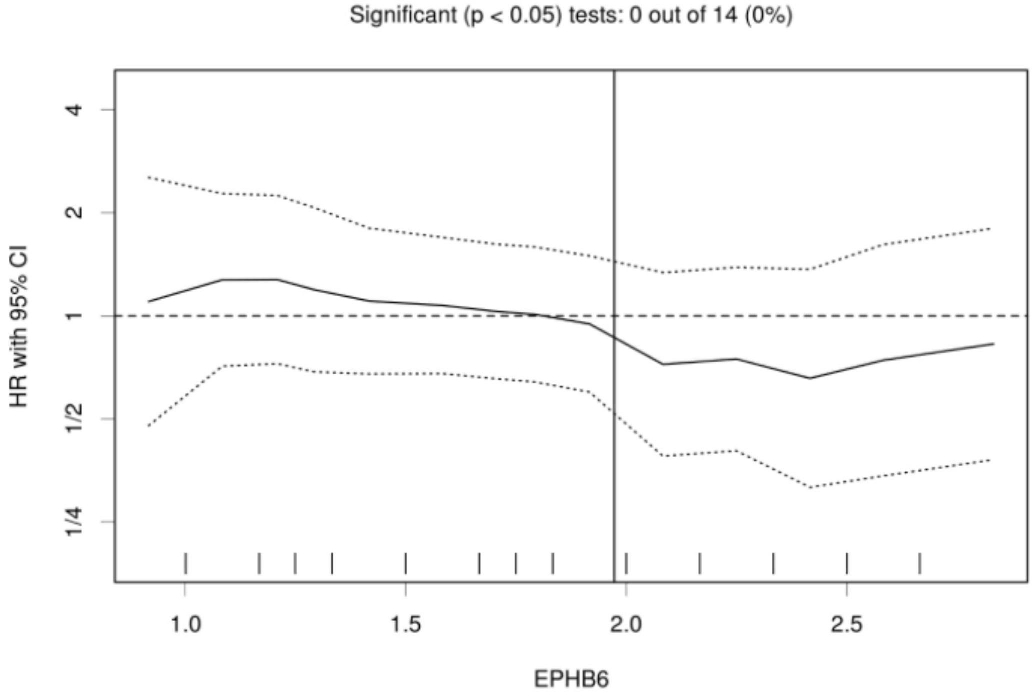

B

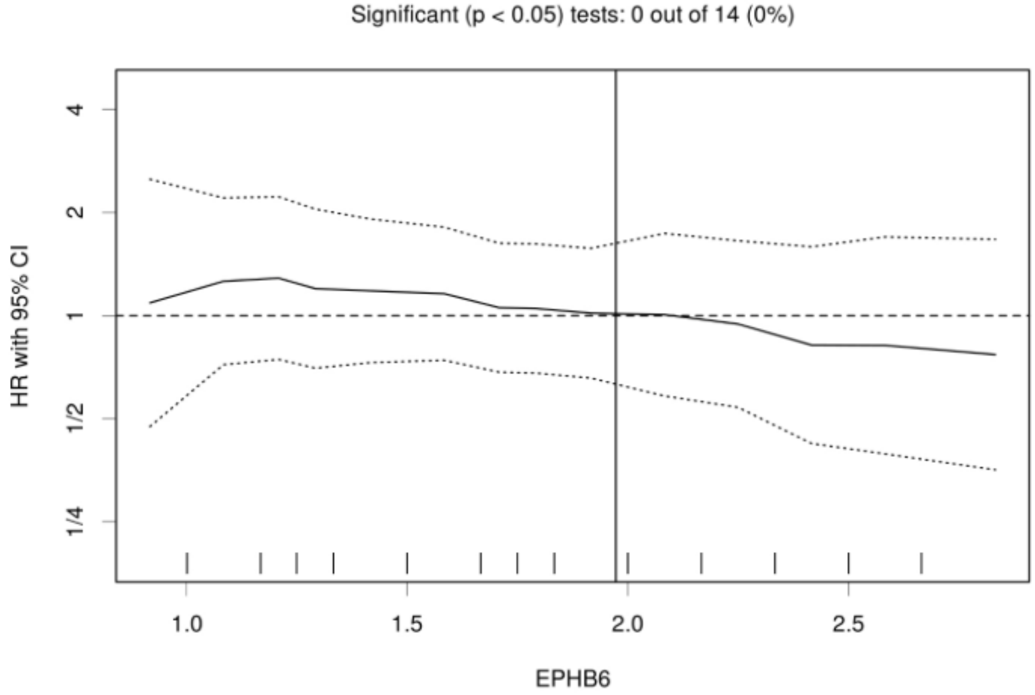

Supplementary Figure 9: Cutoff optimization by correlation with patient survival. For each possible cutoff, EPHB6 expression was correlated with disease-free (A) or overall survival (B). The hazard ratio (HR; solid line) including 95% confidence intervals (dotted lines) is plotted in dependence of the cutoff. A vertical line designates the dichotomization based on the distribution of EPHB6 expression levels in the sample cohort.

Supplementary Table 1: Clinicopathological features of the 130 Dukes C colorectal cancer patients included in the study as a function of EPHB6 expression in their tumors.

	Total	High EPHB6	Low EPHB6	p value
Sex, n (%)				
Female	63 (49.3)	31 (54.4)	32 (45.1)	0.374
Male	65 (50.7)	26 (45.6)	39 (54.9)	
Age (years), mean±SD	66.6	66,65 ± 11,89	66,55 ± 13,07	0.9768
Site, n (%)				
Colon	75 (59.1)	33 (57.9)	42 (60)	0.8572
Rectum	52 (40.9)	24 (42.1)	28 (40)	
Mean follow up (years), mean±SD	9.4	9,4 ± 1,03	9,4 ± 0,90	0.7729
5-year overall survival, n (%)				
Alive	53 (41.5)	23 (40.4)	30 (42.3)	0.8584
Dead	75 (58.5)	34 (59.6)	41 (57.7)	
5-year disease-free survival, n (%)				
Alive	43 (37.1)	20 (40)	23 (34.9)	0.6981
Dead	73 (62.9)	30 (60)	43 (65.1)	
Adjuvant treatment, n (%)				
Yes	49 (41.2)	20 (39.3)	29 (42.7)	0.8509
No	70 (58.8)	31 (60.7)	39 (57.3)	
Microsatellite instability, n (%)				
MSI	16 (12.6)	7 (12.3)	9 (12.9)	1
MSS	111 (87.4)	50 (87.7)	61 (87.1)	
TP53 status, n (%)				
Wild type	18 (46.2)	9 (47.4)	9 (45)	1
Mutant	21 (53.8)	10 (52.6)	11 (55)	
KRAS starus				
Wild type	32 (66.7)	14 (60.9)	18 (72)	0.5427
Mutant	16 (33.3)	9 (39.1)	7 (28)	
Allelic loss of chromosome 18q, n (%)				
LOH	38 (43.7)	18 (44)	20 (43.5)	1
No LOH	49 (56.3)	23 (56)	26 (56.5)	

Figure 1 – FULL LENGTH BLOTS

Original Figure

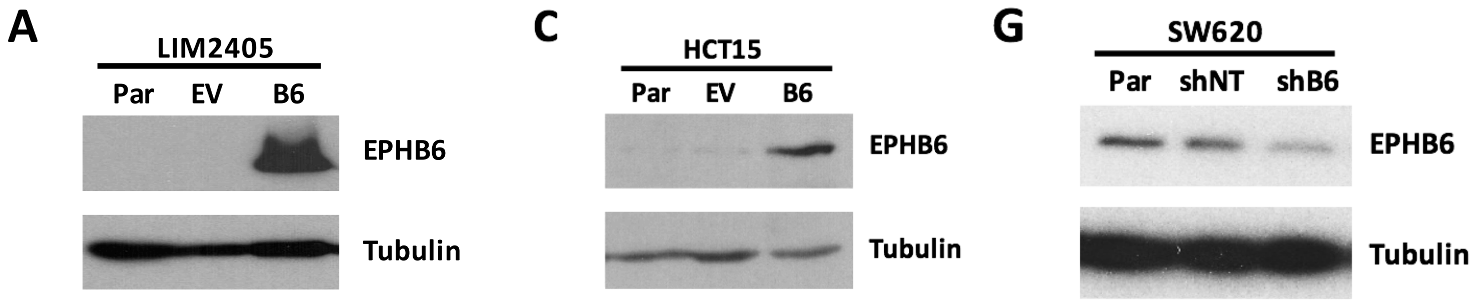

Full length blots

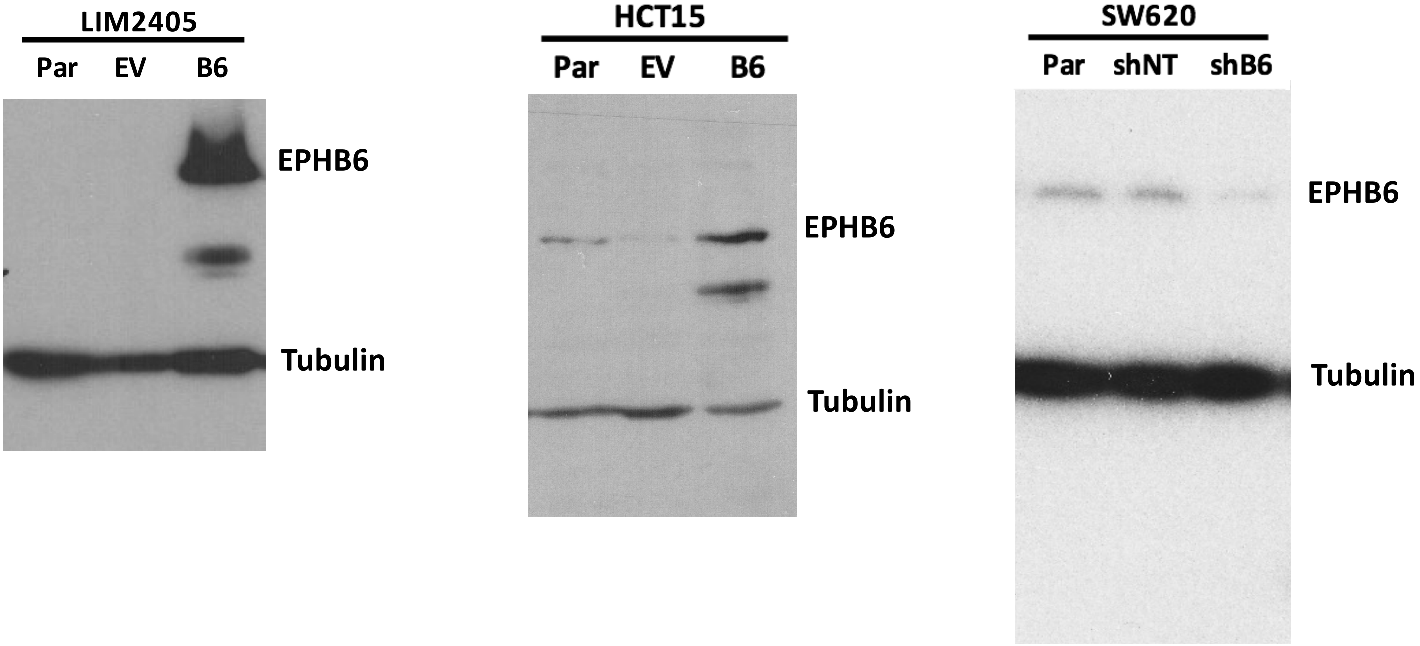

Supplementary Figure 1 – FULL LENGTH BLOTS

Original Figure

A

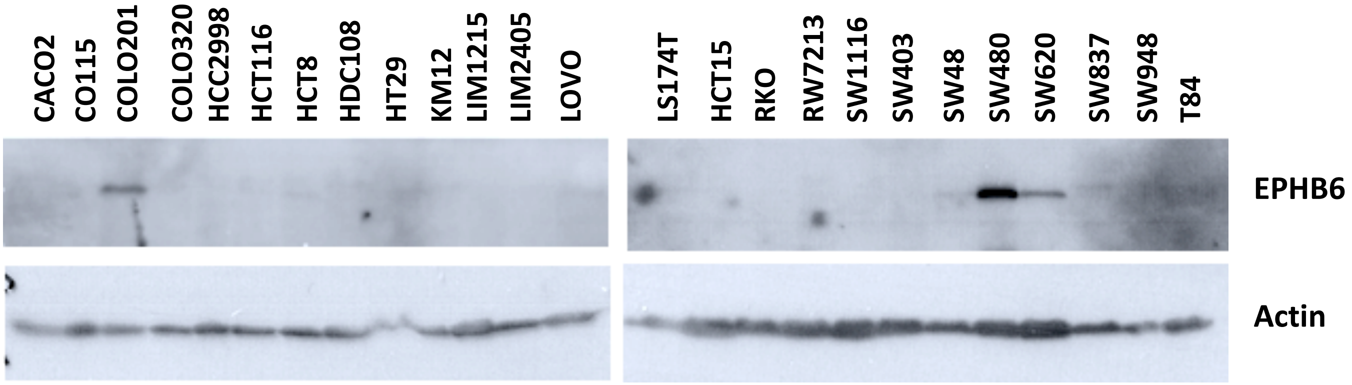

Full length blots

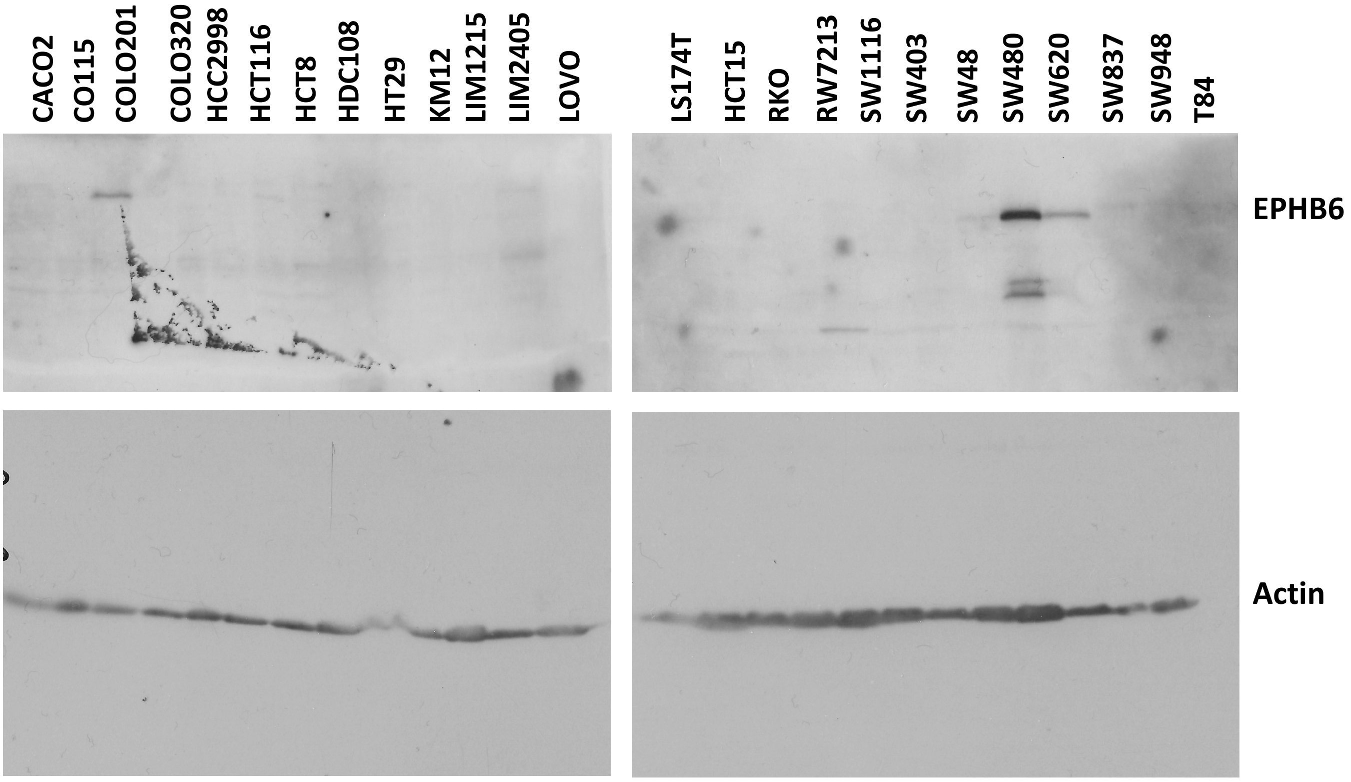

Supplement: Supplementary Materials [file srep43702-s1.pdf]
